# Advances in Plasma and Laser Engineering

**DOI:** 10.3390/ma17081768

**Published:** 2024-04-11

**Authors:** Mariusz Jasiński

**Affiliations:** Institute of Fluid Flow Machinery, Polish Academy of Sciences, Fiszera 14, 80-231 Gdańsk, Poland; mj@imp.gda.pl

## 1. Introduction

Materials science, especially in the context of nanotechnology, plays a key role in today’s world, contributing to the development of advanced materials with unique properties. Plasma- and laser-based techniques have become significant tools, aiding research and application in this field.

This Special Issue of the journal *Materials* is dedicated to describing devices and processes related to advancing plasma and laser engineering. Plasma, recognized as the fourth state of matter, exhibits properties notably distinct from those of ordinary gases. Formed through the ionization of neutral gases, plasma, a conductive gaseous medium, is characterized by a composition of photons, electrons and ions; however, it usually also contains neutral atoms and molecules. The term plasma encompasses media with very different properties, as the composition, densities and kinetic energies of the plasma components vary by several or even more orders of magnitude for different types of plasma. A laser is a device that emits electromagnetic radiation in the visible, ultraviolet or infrared range. It uses the phenomenon of forced emission. Laser radiation is coherent, usually polarized, and has the shape of a beam with very little divergence. In a laser, it is easy to obtain radiation with a very small line width, corresponding to very high power in a selected narrow spectral region. With pulsed lasers, it is possible to obtain very high power in a pulse and a very short pulse duration. Both plasma devices and lasers can have different designs, characteristics and applications. Plasma and laser applications include, but are not limited to, the production of new materials and the enhancement of the properties of existing materials. The plasma or laser treatment of materials can lead to physico-chemical changes in the structure of their surfaces. This Special Issue aims to present advances in plasma and laser technology for different materials.

As the guest editor of this Special Issue of “Advances in Plasma and Laser Engineering” in the “Manufacturing Processes and Systems” section of the journal *Materials*, I will briefly review the general areas of plasma and laser research, including all eight papers published in this Special Issue. Finally, in the Summary, I will propose new challenges as well as new plasma devices and sources of laser radiation for future research in materials science. 

## 2. Results

In the following paragraph, I will briefly describe, in general terms, some selected achievements of plasma technology that are particularly interesting to me. In addition, I will take into account the achievements described by the authors of this Special Issue.

Mankind is at an evolutionary breakthrough in the research and development of plasma techniques, which have become essential for the synthesis and processing of important macro- and nanoscale materials [1]. These materials include nanoparticles, carbon nanotubes and semiconductor nanowires. Plasma processes include the etching and deposition of thin films, catalytic growth of carbon nanotubes and semiconductor nanowires, synthesis of silicon nanoparticles, and the functionalization of carbon nanotubes and self-organizing nanostructures [1,2]. Plasma technologies have applications in fields such as electronics, textiles, welding, automotive, aeronautics, biomedicine and storage devices including batteries, supercapacitors, photocatalysts and electrocatalysts [1,3,4]. Plasma has revolutionized microelectronics, with it being used to deposit and etch thin films in semiconductor devices and integrated circuits [1]. Mankind expects advances in nanoelectronics, photovoltaics, biomedicine and other emerging fields as plasma technology continues to be optimized [1]. 

Alongside the above research areas, research on the plasma etching of silicon and plasma treatment of polyurethane is also presented in this Special Issue. Research on silicone etching was presented by Baek et al. [5] and Krawczyk et al. [6]. Baek et al. in [5] discussed the influence of component ratios on plasma characteristics, active species chemistry and silicon etching kinetics in CF_4_ + O_2_, CHF_3_ + O_2_ and C_4_F_8_ + O_2_ gas mixtures. Plasma diagnostics and etching experiments were performed in the planar inductively coupled plasma reactor. Krawczyk et al., in [6], aimed to modify the surface of silicone rubber using dielectric barrier discharge (DBD) to improve its hydrophilic properties. Research on polyurethane processing was presented by Uricchio et al. in [7]. The authors report the optimization of a two-step atmospheric pressure plasma process to modify the surface properties of a polyurethane foam and, in particular, to produce a superhydrophobic/superoleophilic absorbent for the removal of oils and non-polar organic solvents from water. 

In the paragraph below, I will briefly comment on the selected achievements of laser technology in applications to the synthesis/processing of some materials. 

Lasers have been involved in the production of various types of nanomaterials with improved chemical, optical, magnetic and electronic properties, such as metal nanoparticles, oxides, non-oxides and carbon-based materials [8,9]. Laser technology has enabled the development of photocatalytic and electrocatalytic nanomaterials, among others [8]. Laser techniques such as laser ablation, laser vaporization and laser deposition are used to produce nanoscale materials with a controlled size, shape and specific properties [9,10]. Another technique for the laser processing of nanomaterials that has a major impact on nanotechnology is the laser synthesis and processing of colloids. It is a scalable method for the synthesis of ligand-free nanomaterials in a controlled liquid environment [9,10]. Other applications of laser techniques include the use of a laser beam for cutting, drilling, marking and engraving [11].

The series of articles presented in this Special Issue covers the laser processing of steel and silicon, as well as laser drilling and welding. Maggiore et al., in [12], presented the use of two non-thermodynamic equilibrium surface treatment methods with ultrashort laser pulses to modify the surface of square plates made of austenitic stainless steel. Kovalev et al., in [13], discussed different methods for producing light-trapping “black” silicon, namely laser, chemical and hybrid chemical/laser. Wang et al., in [14], presented the results of a study where a steel plate was welded by ultrasonic-assisted narrow-gap laser welding with a filler wire, and the plasma was observed using a high-speed camera and spectrograph. Fu et al., in [15], presented a research study in which duplex stainless steel was welded by alternating a magnetic field with a laser-arc.

## 3. Summary and Conclusions

The set of eight articles discussed above covers different types of plasmas and lasers, as well as different processes that lead to changes in the properties of different materials. The articles discussed above describe processes such as silicon surface etching, steel surface treatment, laser-plasma welding and the treatment of dielectrics. In addition to experimental results, they include theoretical research and a broad discussion of the phenomena that occur when the plasma or laser beam interacts with material surfaces. These processes have been carried out using different types of plasmas (e.g., inductively coupled plasma, dielectric barrier discharge) and lasers (e.g., 20 ns pumped KrF laser). It seems that the plasma and laser methods presented have potential for implementation in various fields of technology (electrical engineering, electronics, energy, medicine, construction, geodesy, automotive, aviation, shipbuilding, water purification and many other industries). 

I would like to suggest some challenges for the further applications of plasma and laser techniques.

Two-dimensional (2D) materials, such as graphene, are being considered for numerous applications in microelectronics [16,17] and other fields, but their integration into functional devices is challenging [16,18]. In view of the above, several challenges arise. One of the challenges is the poor adhesion of 2D materials due to their chemical inertness [16,19]. I see opportunities here related to plasma and/or laser beam processing. 

In my opinion, the next challenge is to further understand and control the processes that occur when plasma interacts with materials at the nanometer scale. While there is extensive knowledge of the macroscopic effects of using plasma, the understanding of processes at the atomic and molecular level remains limited. Research into the mechanisms of formation and reactions of nanomaterials during exposure to plasma will require interdisciplinary collaboration between scientists from the fields of physics, chemistry and materials engineering. 

In my opinion, an interesting future approach could be the integration of plasma and laser technologies to create advanced nanostructured materials. Using a combination of both technologies can enable the effective synthesis and modification of materials with unique properties that will make a significant contribution to progress in the field of nanotechnology.

Scientists in laboratories around the world are constantly developing new plasma devices and laser radiation sources to achieve unique plasma and laser beam properties. Regarding future research in the field of materials, I would like to suggest the use of the following novel plasma and laser techniques:

- The newest plasma techniques, rarely used so far, include devices for generating plasma planes (e.g., microwave plasma sheets and plasma arrays) and microdischarges (e.g., microwave microdischarges and RF microdischarges), having convenient shapes for materials treatment. So far, only dielectric barrier discharges (DBDs) have been widely used in material surface treatment processes;
- Among the latest laser techniques with a future in materials research, I will suggest picosecond, femtosecond and attosecond lasers (The 2023 Nobel Prize in Physics for the discovery of the possibility of generating attosecond pulses of light, awarded to Pierre Agostini, Ferenc Krausz and Anne L’Huillier!).

## References

[1] Sankaran R.M., Mohan R. (2012). Chapter: Nanoscale Etching and Deposition. Plasma Processing of Nanomaterials.

[2] Steffen T.T., Fontana L.C., Hammer P., Becker D. (2019). Carbon nanotube plasma functionalization: The role of carbon nanotube/maleic anhydride solid premix. Appl. Surf. Sci..

[3] Ouyang B., Zhang Y., Xia X., Rawat R.S., Fan H.J. (2018). A brief review on plasma for synthesis and processing of electrode materials. Mater. Today Nano.

[4] Chokradjaroen C., Wang X., Niu J., Fan T., Saito N. (2022). Fundamentals of solution plasma for advanced materials synthesis. Mater. Today Adv..

[5] Baek S.Y., Efremov A., Bobylev A., Choi G., Kwon K.-H. (2023). On Relationships between Plasma Chemistry and Surface Reaction Kinetics Providing the Etching of Silicon in CF_4_, CHF_3_, and C_4_F_8_ Gases Mixed with Oxygen. Materials.

[6] Krawczyk K., Jankowska A., Młotek M., Ulejczyk B., Kobiela T., Ławniczak-Jabłońska K. (2023). Surface Modification of Silicone by Dielectric Barrier Discharge Plasma. Materials.

[7] Uricchio A., Lasalandra T., Tamborra E.R.G., Caputo G., Mota R.P., Fanelli F. (2022). Atmospheric Pressure Plasma-Treated Polyurethane Foam as Reusable Absorbent for Removal of Oils and Organic Solvents from Water. Materials.

[8] Theerthagiri J., Karuppasamy K., Lee S.J., Shwetharani R., Kim H.-S., Pasha S.K.K., Ashokkumar M., Choi M.Y. (2022). Fundamentals and comprehensive insights on pulsed laser synthesis of advanced materials for diverse photo- and electrocatalytic applications. Light Sci. Appl..

[9] Larosi M.B., García J.V., Rodríguez A.R. (2022). Laser Synthesis of Nanomaterials. Nanomaterials.

[10] Yang L., Wei J., Ma Z., Song P., Ma J., Zhao Y., Huang Z., Zhang M., Yang F., Wang X. (2019). The Fabrication of Micro/Nano Structures by Laser Machining. Nanomaterials.

[11] Sarkar S.K., Tyagi A.K., Ningthoujam R.S. (2022). Lasers in Materials Processing and Synthesis. Handbook on Synthesis Strategies for Advanced Materials.

[12] Maggiore E., Corsaro C., Fazio E., Mirza I., Ripamonti F., Tommasini M., Ossi P.M. (2023). Laser-Treated Steel Surfaces Gliding on Snow at Different Temperatures. Materials.

[13] Kovalev M., Podlesnykh I., Nastulyavichus A., Stsepuro N., Mushkarina I., Platonov P., Terukov E., Abolmasov S., Dunaev A., Akhmatkhanov A. (2023). Efficient Broadband Light-Trapping Structures on Thin-Film Silicon Fabricated by Laser, Chemical and Hybrid Chemical/Laser Treatments. Materials.

[14] Wang R., He Z., Kan X., Li K., Chen F., Fu J., Zhao Y. (2023). Dynamic Characteristics of Plasma in Ultrasonic-Assisted Narrow-Gap Laser Welding with Filler Wire. Materials.

[15] Fu J., Rao Z., Zhao Y., Zou J., Liu X., Pan Y. (2022). Microstructure and Texture Characterization of Duplex Stainless Steel Joints Welded by Alternating Magnetic Field-Assisted Hybrid Laser-GMAW Welding. Materials.

[16] Schätz J., Nayi N., Weber J., Metzke C., Lukas S., Walter J., Schaffus T., Streb F., Reato E., Piacentini A. (2024). Button shear testing for adhesion measurements of 2D materials. Nat. Commun..

[17] Nguyen V.L., Seol M., Kwon J., Lee E.K., Jang W.J., Kim H.W., Liang C., Kang J.H., Park J., Yoo M.S. (2023). Wafer-scale integration of transition metal dichalcogenide field-effect transistors using adhesion lithography. Nat. Electron..

[18] Quellmalz A., Wang X., Sawallich S., Uzlu B., Otto M., Wagner S., Wang Z., Prechtl M., Hartwig O., Luo S. (2021). Large-area integration of two-dimensional materials and their heterostructures by wafer bonding. Nat. Commun..

[19] Lukose R., Lisker M., Akhtar F., Fraschke M., Grabolla T., Mai A., Lukosius M. (2021). Influence of plasma treatment on SiO_2_/Si and Si_3_N_4_/Si substrates for large-scale transfer of grapheme. Sci. Rep..

